# The importance of patient-partnered research in addressing long COVID: Takeaways for biomedical research study design from the RECOVER Initiative’s Mechanistic Pathways taskforce

**DOI:** 10.7554/eLife.86043

**Published:** 2023-09-22

**Authors:** C Kim, Benjamin Chen, Sindhu Mohandas, Jalees Rehman, Zaki A Sherif, K Coombs, RECOVER Mechanistic Pathways Task Force, Boris Julg, Steven B Bradfute, Christian R Gomez, Thomas J Connors, Timothy J Heinrich, W Brian Reeves, Prasanna Jagannathan, Christian Bime, Erin Burke Quinlan, Michael A Portman, Maria Laura Gennaro

**Affiliations:** Infectious Disease Division, Massachusetts General Hospital, Ragon Institute of Mass General, MIT and HarvardBostonUnited States; Center for Global Health, Department of Internal Medicine, University of New Mexico Health Sciences CenterAlbuquerqueUnited States; Division of Lung Diseases, National Institutes of Health (NIH), National Heart Lung and Blood Institute (NHLBI)BethesdaUnited States; Department of Pediatrics, Division of Critical Care, Columbia University Vagelos College of Physicians and Surgeons and New York - Presbyterian Morgan Stanley Children's HospitalNew YorkUnited States; Division of Experimental Medicine, University of California, San FranciscoSan FranciscoUnited States; Department of Medicine, Joe R. and Teresa Lozano Long School of Medicine, University of TexasSan AntonioUnited States; Division of Infectious Diseases and Geographic Medicine, Department of Medicine, Stanford UniversityStanfordUnited States; Division of Pulmonary, Allergy, Critical Care & Sleep Medicine, Department of Medicine, University of Arizona College of MedicineTucsonUnited States; National Center for Complementary and Integrative Health, National Institutes of HealthBethesdaUnited States; Seattle Children's Hospital, Division of Pediatric Cardiology, Department of Pediatrics, University of WashingtonSeattleUnited States; Public Health Research Institute and Department of Medicine, Rutgers New Jersey Medical SchoolNewarkUnited States; 1 Department of Population Health, NYU Grossman School of Medicine New York United States; 2 Division of Infectious Diseases, Department of Medicine, Icahn School of Medicine at Mount Sinai New York United States; 3 Department of Pediatrics, Division of Infectious Diseases, Children’s Hospital Los Angeles, Keck School of Medicine, University of Southern California Los Angeles United States; 4 Department of Biochemistry and Molecular Genetics, University of Illinois, College of Medicine Chicago United States; 5 Department of Biochemistry & Molecular Biology, Howard University College of Medicine Washington United States; 6 Department of Pandemic Equity, Vermont Center for Independent Living Montpelier United States; Augusta University United States; Icahn School of Medicine at Mount Sinai United States

**Keywords:** long COVID, patient-centered, patient-partnered, mechanistic pathways, post-acute sequelae of SARS-CoV-2 (PASC), lived experience, advocacy

## Abstract

The NIH-funded RECOVER study is collecting clinical data on patients who experience a SARS-CoV-2 infection. As patient representatives of the RECOVER Initiative’s Mechanistic Pathways task force, we offer our perspectives on patient motivations for partnering with researchers to obtain results from mechanistic studies. We emphasize the challenges of balancing urgency with scientific rigor. We recognize the importance of such partnerships in addressing post-acute sequelae of SARS-CoV-2 infection (PASC), which includes ‘long COVID,’ through contrasting objective and subjective narratives. Long COVID’s prevalence served as a call to action for patients like us to become actively involved in efforts to understand our condition. Patient-centered and patient-partnered research informs the balance between urgency and robust mechanistic research. Results from collaborating on protocol design, diverse patient inclusion, and awareness of community concerns establish a new precedent in biomedical research study design. With a public health matter as pressing as the long-term complications that can emerge after SARS-CoV-2 infection, considerate and equitable stakeholder involvement is essential to guiding seminal research. Discussions in the RECOVER Mechanistic Pathways task force gave rise to this commentary as well as other review articles on the current scientific understanding of PASC mechanisms.

## Note to the reader

With the goal of engaging a broad audience and connecting medical research directly to patient lived experience, we chose to structure this article in two parallel modes: the more objective (plain text) and the more subjective (italicized quotations). The oscillation between a high-level objective view, and personal, subjective narratives attempts to convey a long hauler’s jarring reality. This structure is analogous to our own experience as patient representatives in the NIH-funded RECOVER research initiative. In this capacity, we find ourselves at the intersection of scientific process and personal need, between supporting the processes of rigorous, inclusive, and nuanced scientific research (which takes time), and desiring solutions for ourselves, our families, and the community at large (which we want as soon as possible). We have been granted a window into the complicated process of developing research studies. We witness expert efforts to address an emergent medical condition about which little is understood, even as we struggle with the day-to-day impacts of this very same condition. All the while, we seek understanding and action from our medical providers, research scientists, government leaders, and social groups. By intermingling the juxtaposing objective and subjective perspectives, we hope to elucidate the experience of the ‘patient representative.’ We use the plural form ‘we’ to indicate our own personal experiences and perspectives as patients, but it also conveys many thoughts of the broader community of long COVID patients with whom we are actively engaging.

- C. Kim and K. Coombs

## Long COVID’s patient community growth followed America’s pandemic response fallout

When the novel coronavirus spread across the globe in the early stages of the SARS-CoV-2 pandemic, people began reporting COVID-19 symptoms persisting beyond the then-expected natural history of two weeks of disease duration. While the emergence of chronically ill patients in past epidemics (Islam et al., 2020) generated little momentum, the sheer numbers of people and repercussions of the SARS-CoV-2 pandemic mobilized masses, including early patients like us. We witnessed the general public’s pandemic fatigue and subsequent increase in high-risk behaviors over time greatly contrasted with our lived experiences. We navigated life-altering illness from a virus that prompted innumerable questions and few answers. Now, chronic symptoms following COVID-19 disease are recognized as post-acute sequelae of SARS-CoV-2 (PASC), and involve ongoing, waxing and waning, or new symptoms, or other health effects occurring after the acute phase of SARS-CoV-2 infection (i.e. present four or more weeks after the acute infection). PASC includes symptoms familiar to many patients as ‘long COVID.’ According to current estimates, such chronic symptoms may affect up to 23 million Americans (U.S. Department of Health and Human Services, 2022a), who also refer to themselves as ‘long haulers.’ Long COVID, a term created by and for patients (Callard and Perego, 2021), is defined as post-infection symptoms or residual illnesses lasting for at least four weeks, endured for a few months to years of progressively severe and debilitating health (U.S. Department of Health and Human Services, 2022b). It may also affect multiple body systems and these systemic manifestations vary over time (U.S. Department of Health and Human Services, 2022b). The prevalence of a persistent post-viral syndrome in such a large patient population could result in an estimated annual loss of 50 billion dollars each year in worker absenteeism (U.S. Department of Health and Human Services, 2022a); a long-term burden on the country’s healthcare system (Maani and Galea, 2020) and a tremendous loss of quality of life. Long COVID is linked to unemployment (Suran, 2023) and may affect up to 16 million working-age Americans (Bach, 2022). Of the estimated 2–4 million people leaving the workforce (Bach, 2022), a significant subset continue to experience persistent long COVID symptoms (Suran, 2023).

In the first two years of the pandemic, we watched mask mandates come and go, vaccines develop and become available, and people isolate and gather again, all while we convalesced intermittently. Despite media reports on new coronavirus variants and the potential to develop long COVID, society’s fatigue with restrictions – in no small part from the whiplash-decision-making (LaFraniere and Weiland, 2022) from political figures and federal agencies – contributed to flouting regulations from the year prior. The clash of affect and reason in risk assessment and resultant desensitization (Slovic and Peters, 2006) toward more than 1.136 million (U.S. Centers for Disease Control and Prevention, 2023) reported American lives lost to date as of July 2023, frustrates and saddens the long COVID community. The growing frustrations of long haulers added to the discouragement from our unattended and overlooked adverse conditions. Some of us acted on our vexation and participated as patient representatives in the RECOVER initiative, part of the National Research Action Plan on Long COVID (U.S. Department of Health and Human Services, 2022a), created from a presidential directive to the Department of Health and Human Services. Some of our drive to become involved with basic research came from being barred from other studies. Many in the ‘first wave’ of the alpha coronavirus variant found themselves unable to participate in research due to stringent protocol criteria: patients’ symptoms would not be recognized as COVID-19-triggered until months later, study time frames could not be changed to include early 2020 SARS-CoV-2-infected patients who did not have a positive antigen test result because testing was scarce at the time, the ICD-10 diagnosis code, U09.9, was released for use well into the pandemic (Pfaff et al., 2023), etc. Regardless of a patient’s ‘study credentials,’ many doctors and researchers were not listening to patients – even when the patients were medical doctors themselves (Yong, 2021).

## Patient motivation to partner with researchers

*K.C., a patient representative from Vermont, learned about long COVID in August 2020 after having symptoms that started in early March 2020 and suddenly worsened. Simultaneously, her two daughters were also experiencing similar health impacts.*

*“I initially became interested in the NIH Recover Study because it included children, and I became an early Caregiver Representative in July 2021 during early brainstorming sessions to begin constructing the RECOVER research initiative.*

*Up until this time, the general world view – including US government and state messaging – had been that children didn't contract SARS-COV-2, or that it was very mild. This had left us out of finding any doctors who had heard of long COVID, and we seemingly continued to fail with our explanations of what this illness looked like for our daughters as often there was no response back to us, as if we had said nothing. It literally felt like no one could hear us, even though we kept trying. I had been seeking support already amongst groups like* Body Politic *and* Long COVID Kids*, and I became more convinced that learning more about long COVID through research could really revolutionize new learning about our immune system as well as the potential to help so many other diseases*.

*I found myself amid a whole large group of researchers, clinicians and other representatives like me who were also concerned; this allowed me to soften, take a breath and realize that after over a full year of my family struggling with debilitating illness while not being believed by practitioners and others, this condition was finally being recognized and* legitimized by the NIH.*”*

## Balancing mechanistic pathways research and long haulers’ needs

Often, the first step toward solving a medical challenge like long COVID comes from understanding the pathophysiology of the disease, COVID-19, or the steps the body endures when perturbed by SARS-CoV-2 (NIAID Funding, 2020). By participating in the RECOVER Mechanistic Pathways committee, we are getting a better sense of the complexity of disease mechanisms, which can include persistence of viral molecules, problems with the immune response, or damage to organs (Chen et al., 2023; Mohandas et al., 2023; Sherif et al., 2022) and can persist for months to years after the initial infection. Understanding why the body does not return to an established, healthy equilibrium will allow for the development of potential therapeutics. Unfortunately, many long COVID patients, ourselves included, remain sick, disabled, and removed from our regular occupations and livelihoods to this day. Waiting for safe and effective mechanistic research to inform solutions takes time. Amidst our own uncertainty and declining health, we became and served as experts, consultants, and advocates, and bridges for both the long COVID sufferers and the biomedical research communities. Intellectual partnerships with efforts like the RECOVER initiative enable learning from each other’s perspective. We help biomedical researchers understand and experience the urgency and impact of their research, as well as the importance of building trust in the community. We also help emphasize the need to build on and complement, rather than merely repeat, existing research from similar conditions, such as ME/CFS, Lyme disease, dysautonomia, and other post-viral conditions (Davis et al., 2023).

In turn, we learn of the complexities that come with merging multidisciplinary fields ranging from basic to applied research, and why researchers focus on understanding mechanisms before rushing into using therapeutics with unclear mechanistic basis or dubious outcomes. For example, while the idea of treating COVID-19 with hydroxychloroquine and ivermectin certainly had misinformation and political influences (Knudsen et al., 2023; Barnett et al., 2022), the underlying paucity of mechanistic basis (Barnett et al., 2022; López-Medina et al., 2021; Mitjà et al., 2021; Self et al., 2020) remains a cautious reminder of false hope and potentially worse health outcomes. Several large, carefully crafted, and compelling research studies have since demonstrated the lack of benefit unproven treatments offer for COVID-19. For example, ivermectin showed no measurable reduction in hospitilization rate (Reis et al., 2022; Bramante et al., 2022) nor severity or timeline of COVID-19 symptoms (Naggie et al., 2022; Lim et al., 2022) even at higher doses and treatment duration (Bibbins-Domingo and Malani, 2023). Similarly, proactively taking hydroxychloroquinine did not prevent COVID-19 (Dhibar et al., 2023) nor reduce mortality (World Health Organization, 2023). In the absence of proven treatments and the midst of confusing information, patient-researcher collaborations help us address the question: how do we balance such urgency with scientific rigor?

## Same fluctuating symptoms on repeat

*“My two daughters and I had multiple new-since-COVID symptoms that kept repeating and disappearing for stretches of time. The recurrence of our initial symptoms along with some new troubling ones made it seem as if we were under attack, and it made me wonder if we were infectious to others, all while my husband continued to remain healthy, which made it all so confusing. We had sore throats, severe stomach aches, headaches, heart palpitations, chest pain, shortness of breath, insomnia, difficulty eating and walking, vision changes, rashes, dizziness, choking throat, painful legs and feet coupled with this new need and desire to lie down, which often brought relief. We would often trend together.*

*When my mother-in-law visited in Aug 2020 and I told her about what was happening, she said to look up long COVID as it sounded similar. We immediately identified with all the other reports that were being covered in the* media (Yong, 2020). *I thought this would be a* unifying (Collins, 2020) *cause everyone could get behind once more people realized that SARS-CoV-2-inflicted disease continued for* some (Lowenstein and Davis, 2021). *We would want to protect each other and learn more so we could begin to start counting SARS-CoV-2 infections more accurately with robust data, and stop the continuation of spread, symptoms and sequelae*.

*RECOVER’s initial patient representative-partnered meetings, Phase 1, included the social determinants of health – promising to put equity first with those who have been most affected by SARS-CoV-2 and recognizing the need to rebuild trust within communities. I was hopeful that RECOVER would raise awareness and there would be media attention and public health messaging to follow. As this is a global problem, the NIH’s recognition of the adult, children and pregnancy cohorts being affected by long COVID could lead by example, and also engage other countries to start acknowledging the declining health in their populations.”*

## A new precedent for patient-centered and patient-partnered research

Historically, healthcare and biomedical research have failed to reflect the diversity of the U.S. population (Bichell, 2015; Cavazzoni et al., 2020; Hermans, 2023). Subsequently, we apply generalizations and assumptions across age, race, ethnic, and social groups despite distinct health or research recruitment needs (Ma et al., 2023; Passmore et al., 2022; Rees et al., 2022). Racism and discrimination in healthcare spans generations and permeates multiple levels, manifesting as chronic stress in an individual (Davies, 2022; Healthy People 2030, 2023), missing public health data points (Krieger, 2021), implicit biases in medical professionals (Davies, 2022), an undiversified healthcare workforce from long-standing discriminatory practices (Blake, 2022; Wilkins et al., 2021), inaccessible medical facilities (Yearby et al., 2022), exclusionary insurance coverage (Artiga et al., 2022; Linke Young, 2020), institutions that perpetuate inequitable systems (Bailey et al., 2017), excessive death and suffering in minority populations (BBC News, 2020; Davies, 2022; Hoffman et al., 2016; Ingraham et al., 2021), and more. Such disparities have persisted with long COVID, disproportionately affecting Brown, Black, Indigenous, and Asian people through higher hospitalization and mortality rates, increased likelihood of developing long COVID and new-onset conditions, and decreased likelihoods of being believed, treated, and diagnosed (Durstenfeld et al., 2023; Jo Hsu, 2022; Khullar et al., 2023; Pfaff et al., 2022; RECOVER Initiative, 2022; Tanne, 2023). Furthermore, when groups often excluded from research or healthcare are incorporated, past mistreatment undermine feeling welcomed and included (BBC News, 2021; Han et al., 2023; Harris, 2016). To address these challenges, healthcare and research can and should increase doctor diversity for improved population health (Snyder et al., 2023), and proactively involve patients at every level of research, from steering committees to community recruitment (Horwitz et al., 2023).

When patients work with researchers to design a large study like RECOVER, having their input contribute to diverse patient recruitment, inclusive eligibility parameters, and accommodations for testing, provides hope that these collaborations represent new precedents in biomedical study design. The RECOVER study has employed a human-centered design (HCD), implementing patient-led research and prioritizing lived experiences (Horwitz et al., 2023; Melles et al., 2021). RECOVER commits to practices like developing culturally responsive and appropriate recruitment materials, communicating findings through seminars like “Understanding Long COVID Across Communities of Color and Those Hit Hardest (RECOVER Initiative, 2022),” and being receptive to the patient community’s emphasis on lived experience by launching projects like the Social, Behavioral, and Economic Impacts of COVID-19 in Vulnerable and Health Disparity Population initiative (National Institutes of Health, 2023). HCD is implemented by many fields such as business and engineering to increase the potential for breakthrough findings. In the context of SARS-COV-2, where nimble yet planned efforts are crucial, HCD encourages patient-centered approaches and guards against repeating research conducted by other post-viral and chronically ill patients with dysautonomia and myalgic encephalomyelitis, or similar phenotypes (Proal and VanElzakker, 2021). Chronic illness patient groups have supported the long COVID community through the uncertainty of new disabilities, the labyrinth of applying for disability benefits, and advocacy efforts. Together, HCD and the solidarity of patient communities create hope that funding may flow toward under-researched areas and that clinical research projects may more actively engage patients as equal stakeholders in the design and implementation.

Healthcare and research efforts are worse off when patients, providers, researchers, or public and private organizations are left in separate silos (White, 2014). The United States’ significant dearth in interoperable data starkly displays the harm of failing to synergize (Mehta and Pandit, 2018). Antiquated, understaffed, and financially constrained, the United States’ public health response abilities are notably deprived. Public health departments largely continue to depend on manual data logging, email, and phone communication. Despite this, their workforce has declined by 15% between 2008 and 2019. Notably, public health makes up a mere 3% of the colossal $3.8 trillion allocated to U.S. healthcare (LaFraniere, 2022). Historic neglect of data interoperability has left a considerable toll, depriving the U.S. of valuable time ahd capabilities during the SARS-CoV-2 pandemic (LaFraniere, 2022). Historic neglect of data interoperability has left a considerable toll, depriving the U.S. of valuable time ahd capabilities during the SARS-COV-2 pandemic (LaFraniere, 2022). Lessons learned from the results of sparse and siloed resources led to the emergence of a new trajectory to advance research. The RECOVER initiative strives to follow this new path by combining efforts, acknowledging the intrinsic worth of every stakeholder.

## Understanding long COVID and assessing need for care

*“I was so inspired by some of the ideas from the early meeting for the RECOVER Initiative in 2021, but at the same time I recognized that a National response would take a long time to launch and most likely would not reach the rural communities near me. I wondered if we could initiate a registry for long COVID cases in Vermont, and specifically within my county. I knew that a wave of illnesses impacted my town in late February/early March 2020, and as Vermont has a lot of small towns, I was concerned there were more individuals experiencing illness like us. Individuals who required comprehensive attention that could benefit from working together to share information and resources. I met with my state senator to see if he could reach out to local doctors and community health leaders to see what they had been experiencing and how to start assessing the need for care. I joined the COVID-19 Longhauler Advocacy Project* (C-19LAP) *in late 2020 and became increasingly interested in learning more about the Vermont Government and doing advocacy work. As individual states began leading the pandemic response within their borders, this work became even more important. I gave my personal narrative at Advocacy Days* (Solve M.E. Advocacy Week, 2021)*, an event in which* Solve ME *included and equipped Longhaulers to share their stories amongst caretakers and people with ME/CFS. We met with Legislative Assistants to US Senators and Representatives in our states hoping to raise awareness. Giving my personal narrative on Advocacy Days in 2021 and 2022 was a powerful experience for me, where I learned about the importance of story and active listening in this new context. I learned that it is ok for me to demand a space to tell my story in whatever way I am comfortable, and that I have things to offer as well as a new outlet for expressing my challenging experiences.”*

## Long COVID’s uncertainty and the freedom of simply asking: why?

The novel coronavirus presents numerous ambiguous challenges, spanning from pinpointing the underlying causes to potential treatments. Acknowledging our lack of answers to various queries can, in a sense, offer a liberating perspective. As early long haulers, it is wearisome to constantly live with waxing and waning symptoms, trying different medications, and navigating an altered life course as we go. All the while, some medical providers feign omnipresent prowess and then offer little resolution. Irrespective of an individual's pre-existing health status, a fundamental question arises: why do some people remain chronically ill? How does this virus affect seemingly every organ system and cause so many disabilities? Conversely, the question arises as to why certain individuals effectively eliminate the infection without symptoms or significant complications. Could it be the host genetics that is implicated in differential immunological responses to infection and disease progression (Augusto et al., 2023)? We anticipate the RECOVER initiative, along with other biomedical researchers, will shed light on these inquiries and more. Partnering with researchers helps us reframe the endless uncertainty to endless potential answers. Investigating possible mechanistic pathways to find explanations, or actively searching for explanations in uncertain times, can reduce fear and offer hope to patients in finding resolutions and the next steps.

## Inability to participate in research and long COVID clinics

*“Our story is about the fight for legitimacy and validity as patients seeking care, a status that has remained elusive throughout the epidemic and pandemic phases. With my past work as an acupuncturist, I have listened to many reports from patients about barriers to receiving effective Western medical care. I also decided to go to acupuncture school in 1997 after becoming sick from a parasitic infection, Ascaris. I started having constant headaches and nausea and sought out an acupuncturist then. It wasn't a magic cure in the sense that I was suddenly better – as it took many more years to fully recover – but I did begin a new love for this type of medicine, as I felt less turmoil and more ease internally with Chinese Medicine in a way that I hadn’t experienced before, all while learning new ways to interpret health and disease.*

*Years later, soon after moving to Vermont, one of my daughters became sick with acute to chronic Lyme disease when she was 6, and it took 1.5 years to recover. My family learned that illness has its own timeline, regardless of how much time is spent chasing doctors and trying hard to get better. We were left to figure it out mostly on our own, and I am certain that this second hit from SARS-CoV-2 was exactly what my daughter didn't need, even with being well for 2 years in between.*

*It has risen to another level of difficulty to be on the other side of wellness, with 3 out of 4 of our immediate family becoming suddenly chronically ill and disabled from a new airborne virus, while few doctors have been trained to recognize or treat post viral illnesses; it has been an impossible task on every front as complex, invisible diseases are too easily dismissed in Western medicine.*

*The lack of testing and knowledge at the beginning of the pandemic really harmed our ability to seek care later with any credibility. When we began seeking guidance for our various emerging symptoms, I made sure to talk about SARS-CoV-2 and long COVID at every medical appointment with hopes to get this information in our doctor’s notes from the beginning. I divided up our medical needs into what were our top 3 most debilitating problems, and sought out referrals to those specialists. Three and a half years after our initial onset of continual symptoms, we are still working on making a team for each of us that is respectful and supportive while we continue to wait for more answers and therapeutics.”*

## Existing challenges to PASC

While basic research is underway, multiple challenges to PASC remain. For example, limited public understanding, inconsistent and highly demanded disability support, and complicated ways to translate scientific findings (COVID-19 Longhauler Advocacy Project, 2022; Department of Health and Human Services, 2022; McCorkell, 2023; OPEN LETTER | COVID-19 Longhauler Advocacy Project | C-19 LAP, 2022). From May 21 to June 10, 2021, a survey of nearly 2000 American adults found that over 30% of Americans were unaware of long COVID and 39% remained unconcerned after reading a description of the condition (Resolve to Save Lives, V. S, 2021). On a global level, Google search trends for the term ‘COVID-19’ dominated online interest in 2020 and yielded significant interest since then, suggesting common awareness of SARS-CoV-2, but far smaller search interest in ‘long COVID’ and related terms (Kaatz et al., 2022). Despite immense PASC patient numbers, the far-reaching impacts of long COVID (U.S. Department of Health and Human Services, 2022a), and general awareness of the condition’s catalyst, a significant portion of the general populace is not aware or attentive to this issue. This could reduce the likelihood of public health communication within communities and common knowledge of pertinent research studies, such as RECOVER, that may be recruiting locally.

For those living with PASC, despite awareness of the condition and possible knowledge of research studies, the need to acquire disability support often takes precedence. The impact of disability support for long haulers is significantly influenced by their social determinants of health (Social Determinants of Health, 2022), especially the structural and individual discriminatory conditions like housing and education segregation, racism, and ableism, that affect resource access. America’s already strained social safety net (Rowland, 2022) with claim approval dependent on meeting documented loss of function criteria, often in the form of abnormal test results, means many newly disabled long haulers struggle to obtain financial benefits and workplace accommodations (Stead Sellers, 2022). As of March 2022, the Social Security Administration received approximately 23,000 disability applications related to COVID (Rowland, 2022), representing a fraction of 1% of total annual applications. In 2020 and 2021, Social Security’s Disability program received 1,838,893 and 1,820,282 applications for disabled-worker benefits, respectively, approving less than 36% of all applications each year; a declining approval trend since 2009 (U.S. Society Security Administration, 2022). The demand for existing benefits among existing disabled and chronically ill populations (Stead Sellers, 2022) only increased with the pandemic’s onset, leaving many long haulers in uncertain financial and medical situations. Additionally, America’s laws reside in health, education, and workforce sector silos (Sperling, 2020; Wagner Mery, 2022) that can delay a patient’s journey to obtain necessary support and resources by years (Wagner Mery, 2022). While experts and experienced disability advocates view the long COVID community as a potential changemaker in America’s disability infrastructure (Stead Sellers, 2022) due to sheer population prevalence, these changes will take time.

Time may be the most acutely felt pain for patients, providers, and researchers in addressing long COVID. Support for basic research on long COVID in America is moving slowly (Wadman, 2022) and with aversion to risk (Kaiser, 2022), as the National Institutes of Health aims to carefully balance speed and scientific rigor to allocate taxpayer money into research grants that return on investment. While other countries such as the United Kingdom moved swiftly to fund 15 long COVID clinical trials by July 2021 (Wadman, 2022), as of February 2022, the United States had only funded 8 of over 200 long COVID clinical trials (The Rockefeller Foundation et al., 2022). As scientific institutions weigh research best practices, data-driven healthcare, and treatment urgency to clarify optimal patient care, caregivers and patients experience a stressful reality. Patients desperate for symptom relief who have extra resources and time are turning to the growing market of businesses and private practices with mixed results. Many go through trial and error with treatments; an experience akin to guessing as treating long COVID may require tailored care to a specific phenotype (Shaffer, 2022). Anecdotally from online patient group discussions, those willing and able to try unsubstantiated treatments report variable degrees of relief or symptom progressions based on inumerable factors. Groups that allow sharing of medication or treatment information often emphasize the patient experience and the supervision of a doctor. However, as a result of many companies taking advantage of patients and profiteering from the mass suffering of long COVID, other groups are strictly moderated and prohibit treatment discussion.

Further exacerbating PASC’s time pressure is the NIH’s historic underfunding of overlapping illnesses with potential post-viral origins that have extremely similar phenotypes to long COVID (Komaroff, 2019). If research for diseases such as myalgic encephalomyelitis/chronic fatigue syndrome (ME/CFS), which affects an estimated 1–2.5 million Americans as of 2020 (Mirin et al., 2020), was appropriately funded in years past according to disease burden (Green et al., 2015; Mirin et al., 2020), we would likely have more insight and progress on addressing long COVID today. Long COVID patient advocacy grew under the guidance of and commonalities with existing chronic illness communities. Chronic underfunding of ME/CFS (Mirin et al., 2020) and other post-viral illness research such as dysautonomias, as well as neglecting existing research on such topics (Proal and VanElzakker, 2021), undermines efforts to address long COVID. The slow pace of funding and establishing foundational research for post-viral illnesses and RECOVER’s focus on data collection concerns patients and doctors that time is not being used efficiently. While clinical trials for therapeutics are in the works, their pace feels especially slow, knowing private-public partnerships such as those seen in developing COVID-19 vaccines (Cohrs, 2022) can be nimble and ground-breaking. We look forward to mechanistic research on COVID-19 building a strong foundation for therapeutics to build on, but in the meantime, we must live with new disabilities and different qualities of life. Constructing and translating PASC research, balancing scientific rigor and legitimate stakeholder concerns, and fielding low public awareness and disability support for long COVID unfortunately give rise to the perception that we are building the plane as it is moving down the runway.

## Patient-partnered research is humanizing and meaningful

*“There were some days where I wondered who I was after becoming a COVID long hauler in early 2020. For months, I lost the ability to walk, smell, breathe, read, write, communicate, cook, and sleep. Medical providers eventually diagnosed me with COVID-19-triggered functional disorders and syndromes that many tests failed to capture as abnormal. Instead of new adventures soon after graduating college, I had my 24th birthday alone in a walker and was terminated from my first job as an engineer. I realized I valued myself based on my skills and my identities, when long COVID took so many and I didn’t know what I had left. For some time, I couldn’t name what I was good at or what I liked because my numerous new disabilities required me to drastically change my lifestyle. Over three years after my initial April 2020 infection, I am still disabled and live with significant changes in my daily life. I have experienced multiple symptom fluctuations frustrations, difficult side effects from treatments, and am now at a place where I am tired of trying potential cures. I find it more worth my time and effort to focus on accessibility, accommodations, and the human, rather than the technical, side. While I am not back to my previous functioning and still look forward to future therapeutics that may ease suffering from long COVID, partnering with RECOVER to support patient-centered mechanistic research feels meaningful. – C.K.”*

## Future impact

While significant challenges to understanding and treating long COVID exist, such as low public and medical awareness, historic racial disparities in healthcare access, chronic underfunding of post viral illness research, and time urgency, collaborative and mutually beneficial patient-provider-researcher relationships can play a central role in achieving success. For example, grassroots, patient-partnered organizations and research groups have garnered increased awareness of postviral conditions in communities of color and greater attention to ameliorating racial biases in healthcare (Bright Star Community Outreach, 2023; C19LAP, 2023; Dysautonomia International, 2023; Illinois Unidos, 2023; Jason and Torres, 2022; Long-COVID Alliance, 2023; Massachussetts General Hospital, 2022; #MEAction, 2023; PLRC, 2023; [Bibr bib82][Bibr bib87]; The Ehlers-Danlos Society, 2023). The importance of long COVID has also been reflected in proposed legislation, a National Academies committee to examine the working definition, and a HHS long COVID report naming racial disparities as a key obstacle to understanding and treating long COVID (Department of Health and Human Services, n.d.; Eldahshoury, 2023; National Academies of Science, Enginering, and Medicine, 2023; Kaine, 2023). This in turn has contributed to meaningful efforts in addressing the racial disparity of rare and chronic postviral-associated diagnoses in people of color (dforsythe, 2020), particularly following long COVID (Khullar et al., 2023). Experts recognize that the typical approach to describing a new disease based on a constellation of symptoms is not fruitful for understanding long COVID (Cooney, 2022). Expertise on long COVID falls within the patient realm as well. Research institutions are challenged with a new precedent following the COVID-19 pandemic to equitably partner with patients in research efforts. We look forward to RECOVER and other biomedical studies contributing to an increased understanding of and potential treatments for PASC. Investing in long COVID and related post-viral illness research in concert with sustainable accessibility support for disabled and chronically ill patients will result in long-term benefits for populations with unmet needs beyond long COVID patients.

## A note from the medical co-authors

In the fight against HIV/AIDS, Dr. Anthony Fauci has acknowledged the central role that patient activists played in pushing for faster and innovative trial designs when they demanded a seat at the table. Similarly, in the fight against long COVID, the engagement of patient representatives as research partners is needed to press for systematic trials and to help focus research on involving patients in much needed trials. Partnerships with patient advocacy groups are also needed to communicate the urgent need for resources that improve quality of life until more definitive treatments for long COVID are identified.
